# Silicon-Enriched Meat Ameliorates Diabetic Dyslipidemia by Improving Cholesterol, Bile Acid Metabolism and Ileal Barrier Integrity in Rats with Late-Stage Type 2 Diabetes

**DOI:** 10.3390/ijms252111405

**Published:** 2024-10-23

**Authors:** Marina Hernández-Martín, Alba Garcimartín, Aránzazu Bocanegra, Adrián Macho-González, Rosa A. García-Fernández, Sonia de Pascual-Teresa, Rocío Redondo-Castillejo, Sara Bastida, Francisco J. Sánchez-Muniz, Juana Benedí, Mª Elvira López-Oliva

**Affiliations:** 1 Departmental Section of Physiology, Pharmacy School, Complutense University of Madrid, 28040 Madrid, Spain; marinh04@ucm.es; 2 AFUSAN Research Group, Sanitary Research Institute of the San Carlos Clinical Hospital (IdISSC), 28040 Madrid, Spain; a.garcimartin@ucm.es (A.G.); aranboca@ucm.es (A.B.); amacho@ucm.es (A.M.-G.); roredond@ucm.es (R.R.-C.); sbastida@ucm.es (S.B.); frasan@ucm.es (F.J.S.-M.); jbenedi@ucm.es (J.B.); 3 Pharmacology, Pharmacognosy and Botany Department, Pharmacy School, Complutense University of Madrid, 28040 Madrid, Spain; 4 Nutrition and Food Science Department, Pharmacy School, Complutense University of Madrid, 28040 Madrid, Spain; 5 Animal Medicine and Surgery Department, Veterinary School, Complutense University of Madrid, 28040 Madrid, Spain; ragarcia@ucm.es; 6 Department of Metabolism and Nutrition, Institute of Food Science, Technology and Nutrition (ICTAN-CSIC), 28040 Madrid, Spain; s.depascualteresa@csic.es

**Keywords:** cholesterol-lowering agent, bile acids and cholesterol metabolism, diabetic dyslipidemia, silicon-meat functional food, intestinal barrier integrity

## Abstract

Silicon as a functional ingredient of restructured meat (RM) shows antidiabetic and hypocholesterolemic effects in a type 2 diabetes mellitus (T2DM) rat model. The present paper investigated the mechanisms involved in this cholesterol-lowering effect by studying the impact of silicon-RM consumption on bile acid (BA) and cholesterol metabolism. In addition, the main effects of cecal BA and short-chain fatty acids derived from the microbiota on intestinal barrier integrity were also tested. Rats were fed an RM high-saturated-fat, high-cholesterol diet (HSFHCD) combined with a low dose of streptozotocin plus nicotinamide injection (LD group) and for an 8 wk. period. Silicon-RM was included in the HSFHCD as a functional food (LD-Si group). An early-stage T2DM group fed a high-saturated-fat diet (ED group) was used as a reference. Silicon decreased the BA pool with a higher hydrophilic BA profile and a lower ability to digest fat and decreased the damaging effects, increasing the occludin levels and the integrity of the intestinal barrier. The ileal BA uptake and hepatic BA synthesis through CYP7A1 were reduced by FXR/FGF15 signaling activation. The silicon up-regulated the hepatic and ileal FXR and LXRα/β, improving transintestinal cholesterol (TICE), biliary BA and cholesterol effluxes. The inclusion of silicon in meat products could be used as a new therapeutic nutritional tool in the treatment of diabetic dyslipidemia.

## 1. Introduction

Type 2 diabetes mellitus (T2DM) is a chronic disease characterized by hyperglycemia subsequent to insulin resistance and β cell dysfunction [1]. T2DM is a serious and global public health problem, and it is estimated that by 2035, there will be more than 590 million people diagnosed with T2DM [2]. There is a direct relationship between hyperglycemia and plasma lipid elevation, explaining why dyslipidemia is extremely common in T2DM [3]. Diabetic dyslipidemia is characterized by increased total cholesterol and triglyceride levels, with important alterations in the lipoprotein profile [4]. Pharmacological and nutritional interventions aimed at restoring cholesterol homeostasis are based on the control of its biosynthesis, absorption and efflux, transport, storage, use and excretion [5]. The catabolism of cholesterol to bile acids (BAs) is the predominant mechanism eliminating excess cholesterol from the body. BAs play critical roles in regulating and maintaining lipid, glucose and energy metabolisms, subsequently protecting against inflammation in the liver, intestine and heart [6]. The link between diabetic dyslipidemia and the dysregulation of BA metabolism has been suggested [7]. Targeting BA signaling pathways, as the main cholesterol excretion pathways, has emerged as a promising therapeutic strategy for the management of diabetic dyslipidemia. Changes in total BA concentrations or their profile and an increase in the hydrophobic/hydrophilic BA ratio have been closely associated with the development and progression of T2DM [8,9,10]. In this regard, BA sequestrant agents decrease the amount of BA returning to the liver through the enterohepatic cycle, and thus stimulate the conversion of cholesterol to BA, which may improve glycemia and cholesterolemia [11]. It is well known that the release of the ileal fibroblast growth factor 19/15 (FGF19/15), induced by the farnesoid X receptor (FXR), leads to a reduction in BA production by suppressing the expression of CYP7A1/CYP8B1 in the liver [6]. Furthermore, FXR inhibits the hepatic uptake of BA by the Na^+^-taurocholate co-transporting polypeptide (NTCP) [12], as well as increases the functions of the hepatic bile salt export pump (BSEP) [13], resulting in a higher excretion of BA. Hence, the nutritional or pharmacological strategies that lead to FXR activation promote insulin sensitivity [11] and consequently improve glycolipid metabolism [14].

In addition, a high-saturated-fat diet (HSFD) and, specifically, the excessive consumption of red and/or processed fatty meats, common in the Western diet pattern, have been proposed as inducers of dyslipidemia and proposed to be a pathogenic risk factor for T2DM [15]. The intake of these atherogenic diets has been associated with changes in BA metabolism [16]. Fecal 12 α-hydroxylated BA (12α-OHBA) increases have been positively associated with glucose intolerance in HSFD-fed rats [17] and may play a role in the progression of T2DM [18]. A high-saturated-fat, high-cholesterol diet (HSFHCD) may also alter the gut microbiota, causing secretory changes in intestinal microbial metabolites, such as BA and short-chain fatty acids (SCFAs), triggering a series of possible mechanisms that lead to T2DM [19,20]. The altered BA pool and its metabolites are inherently cytotoxic, and their effects transcend local intestinal inflammatory processes or low-grade systemic inflammation due to increased intestinal permeability, leading to hepatic metabolic disorders.

There is strong evidence that the consumption of cholesterol-lowering agents, either as dietary supplements or as functional food ingredients, is an alternative treatment for diabetic dyslipidemia [21,22]. Some functional ingredients interfere with BA reabsorption, increasing its fecal excretion. These ingredients could inhibit the conversion of primary bile acids (PBAs) to secondary bile acids (SBAs), thereby reducing the intestinal absorption of fat and cholesterol [23]. Functional foods could be used to provide an additional lipid-lowering effect and to reduce BA accumulation, thereby preventing the need for pharmacotherapy or allowing a decrease in medication doses [24,25]. Specifically, the design of new functional meat formulations that make meat a healthier food and that are used as a vehicle for the inclusion of bioactive ingredients could be a therapeutic strategy for the treatment of T2DM. Functional meats can also be a complementary therapeutic tool in patients with low adherence to medication.

Our research group has extensive experience in the design and development of meat-based functional foods with lipid-lowering capacities [26,27,28,29,30,31,32,33,34,35,36]. Previous studies have demonstrated the ability of silicon to improve insulin sensitivity. They hypothesized that it could influence insulin signaling pathways, enhancing their actions and facilitating glucose uptake by cells, which supports their hypoglycemic effects. Likewise, silicon is able to increase the expression and activity of intestinal glucose transporters, thereby promoting the reduction of postprandial glycemia [37]. We have already demonstrated that the consumption of a silicon-enriched functional meat (Si-RM) has hypoglycemic and hypolipidemic effects, improving the lipoprotein profile [29,33,34,35] by inhibiting the absorption and increasing the efflux of cholesterol in the proximal small intestine [33,36]. As a refinement and extension of our previous work, the current study is dedicated to delving into the mechanisms by which the consumption of Si-RM ameliorates diabetic dyslipidemia by restoring the BA metabolism in rats with late-stage T2DM. Concretely, our aim is based on the evaluation of silicon effects on the cecal BA and SFCA contents and profiles, cholesterol and BA synthesis, the absorption or excretion molecular mechanisms, as well as the impact of silicon on ileal barrier integrity.

## 2. Results

### 2.1. Ponderal Parameters, Amounts of Fecal Fat and Cholesterol Excretion and Cecal Mucosae and Fecal Silicon Contents

No significant differences in daily total intake, body weight increase and small intestine and cecum weights were found between groups (*p* > 0.05) (Table 1) (ANOVA, Bonferroni post hoc test, *p* < 0.05). Liver weight was higher in the LD (78.0%) and LD-Si (68.5%) groups compared to their ED counterpart (*p* < 0.001). There was a tendency toward lower liver weight in LD-Si rats with respect to LD, but without significant differences.

Significant differences were observed in cholesterol intake, fecal excretion, fecal fat and cholesterol amounts (*p* < 0.0001). Cholesterol intake was higher in the LD and LD-Si groups compared to the ED group (*p* < 0.0001). In LD rats, fecal excretion (40.6%), fecal fat (112.3%) and fecal cholesterol (213.5%) excretions were significantly higher (*p* < 0.05) compared to ED rats. The LD-Si group showed significantly higher total fecal excretion (22.1%), fecal fat (49.5%) and cholesterol (68%) excretions (*p* < 0.01) than the LD group. When compared to the ED group, LD-Si rats exhibited the highest fecal excretion (70.6%) and fecal fat content (217.5%) *(p* < 0.01).

Fecal silicon content was lower in LD rats compared with ED. Si-RM consumption significantly increased the fecal silicon content by about 117.6% (*p* > 0.0001) with respect to LD, achieving ED levels (*p* > 0.05). The daily silicon excretion increased in LD-Si rats by 118.83% (*p* < 0.0001) with respect to ED and 135.1% (*p* < 0.0001) with respect to the LD group. Silicon content in the cecal mucosa was lower in LD (−33.5%, *p* < 0.001) and LD-Si (41.2%. *p* < 0.001) groups compared to the ED group. No significant differences between LD and LD-Si rats in cecal silicon content were found (*p* > 0.05) (Table 1).

### 2.2. Blood Glucolipid Metabolism

Glycemia, insulinemia, HOMA-IR, HOMA-β, triglyceridemia, cholesterolemia, atherogenic index (AI), diabetes trend score (DTscore) and dyslipemic diabetes score (DDscore) are shown in Table 2 (ANOVA, Bonferroni post hoc test, *p* < 0.05).

The results revealed significant differences in all the parameters displayed in Table 2 (*p* < 0.001). LD rats presented significantly higher levels of glycemia (*p* < 0.001) but lower levels of insulinemia and HOMA indexes (*p* < 0.001) compared to ED rats. LD-Si rats showed intermediate and significantly different values for all parameters (*p* < 0.01) compared to LD (except for HOMA-IR), which did not show significant differences between them and ED rats (except for glycemia, where no significant differences were observed) (Table 2). All groups displayed high plasma glucose levels (≥11.1 mmol/L), confirming T2DM. Plasma triglycerides, cholesterol and AI (total cholesterol/HDLc) were significantly affected by the diets (*p* < 0.01) (Table 2). Hypercholesterolemia was prevalent in LD rats (87.5%) but absent in ED and LD-Si rats. The LD group showed higher AI compared to the ED group (*p* < 0.001). DTscore and DDscore showed significant changes among the three experimental groups (*p* < 0.001). LD rats showed a significantly higher DTscore and DDscore than ED (*p* < 0.001). The LD-Si group showed a significantly higher DTscore with respect to ED (86.6%, *p* < 0.001) but lower with respect to the LD group (28.3% *p* < 0.001). LD-Si rats also showed a lower DDscore with respect to the LD group, and no significant differences were found with respect to ED animals (*p* < 0.05) (Table 2). These results indicate that Si-RM consumption improved plasma diabetic dyslipidemia parameters observed in the LD rats.

### 2.3. Cecum Bile Acid Composition and Profile

**Figure 1:**
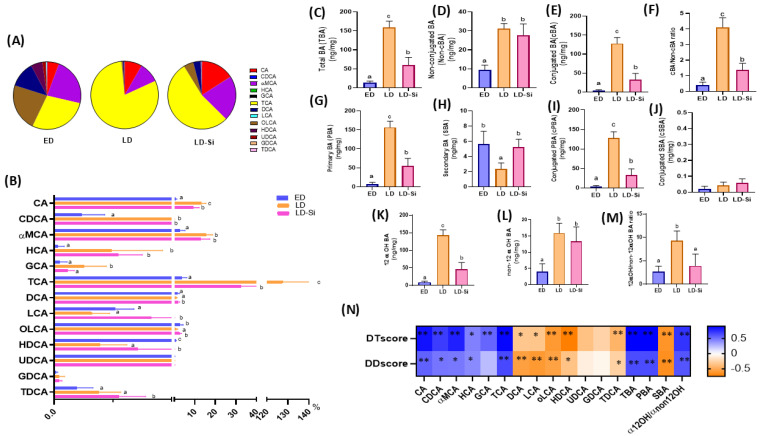
Cecal bile acids (BA) contents and profile in the early-stage diabetes (ED), late-stage diabetes (LD), and late-stage diabetes-silicon (LD-Si) groups. BA content was expressed in ng/mg. Values expressed as mean ± SD. Different letters (a < b < c) indicate significant differences between groups (ANOVA, Bonferroni post hoc test, *p* < 0.05). (**A**) BA percentages in each experimental group, (**B**) BA profile, (**C**) Total bile acids (TBA), (**D**) Non-conjugated BA (non-cBA), (**E**) Conjugated BA or Bile salts (cBA), (**F**) Conjugated/non-conjugated BA ratio (cBA/non-cBA), (**G**) Primary BA (PBA), (**H**) Secondary BA (SBA), (**I**) Conjugated PBA (cPBA), (**J**) conjugated SBA (cSBA), (**K**) 12 α-hydroxylated BA (12α-OHBA), (**L**) non-12 α-hydroxylated BA (non-12α-OHBA), (**M**) 12α-OHBA/non-12α-OHBA ratio, (**N**) Heatmap showing relation between BA profile and DTscore and DDscore. The color intensity of the heatmap represents the association degree: Blue, positive association; Orange, negative. * Denotes adjusted: * *p* < 0.01, ** *p* < 0.001. Pearson’s correlation coefficients values were used for the matrix.

Thirteen free BAs were detected in the cecal contents (Figure 1A,B). Four of them (cholic acid (CA), chenodeoxycholic acid (CDCA), α-muricolic acid (α-MCA) and hyocholic acid (HCA)) were primary bile acids (PBAs), with two conjugated forms of CA (cPBA) (taurocholic acid (TCA) and glycolic acid (GCA)); and five (deoxycholic acid (DCA), lithocholic acid (LCA), oxo-lithocholic acid (oLCA), hyo-deoxycholic acid (HDCA) and urso-deoxycholic acid (UDCA)) were secondary bile acids (SBAs), with the two conjugated forms of DCA (cSBA), tauro-deoxycholic acid (TDCA) and glyco-deoxycholic acid (GDCA). In addition, Figure 1C–M presents the results obtained from the sum of the contents of: (C) total bile acids (TBAs), (D) non-conjugated BA, (E) conjugated BA or bile salts, (F) conjugated/non-conjugated BA ratio, (G) primary BA (PBA), (H) secondary BA (SBA), (I) conjugated PBA (cPBA), (J) conjugated SBA (cSBA), (K) 12 α-hydroxylated BA (12α-OHBA), (L) non-12 α-hydroxylated BA (non-12α-OHBA) and (M) 12α-OHBA/non-12α-OHBA ratio (ANOVA, Bonferroni post hoc test, *p* < 0.05).

The BA composition of the three experimental groups expressed as a percentage of each BA relative to TBA is shown in Figure 1A. The most abundant BAs (percentages > 5.0%) in the pool were as follows: ED group: TCA (28.49%), αMCA (22.91%), oLCA (22.73%), DCA (12.29%), HDCA (5.56%) and CA (5.50%); LD group: TCA (80.33%), αMCA (9.75%) and CA (8.28%) and LD-Si group: TCA (53.81%), αMCA (21.46%), CA (15.69%), oLCA (4.69%) and DCA (3.46%).

The individual BA profiles, from highest to lowest concentrations in the cecum of the three experimental groups, are shown in Figure 1A,B. Specifically, in ED rats, the highest BA contents (>0.5 ng/mg) were TCA > αMCA > oLCA > CDA > HDCA > CA, while both LD and LD-Si groups followed the order of TCA > αMCA > CA > oLCA > CDA > UDCA > HDCA, demonstrating that Si-RM consumption induced minimal changes in the BA profile compared to HSFHCD (Figure 1A). Regarding the individual BA amounts (Figure 1B), the LD group showed higher CA, CDCA, α-MCA, HCA, GCA and TCA contents (ranged from 5.00- to 34.38-fold) and lower HDCA (18.46-fold) and oLCA (2.60-fold) contents (*p* < 0.05) compared to those of ED rats, with no changes in DCA, LCA, UDCA, TCDA and GCDA (*p* > 0.05). LD-Si rats displayed higher levels of CA, CDCA, αMCA, HCA, TCA, DCA, GDA, TDA, GDCA, TDCA (which ranged from 2.75- to 12.6-fold, *p* < 0.0001) compared to ED rats, with no changes in DCA, LCA, oLCA, HDCA and UDCA (*p* > 0.05). Compared to LD rats, silicon significantly decreased CA (1.39-fold, *p* < 0.01) and TCA (3.91-fold, *p* = 0.001) and increased DCA, LCA, oLCA contents (which ranged from 2.48- to 2.71-fold, *p* < 0.001). No significant differences were found in CDCA, αMCA, HCA, GCA, UDCA, GDCA and TDGA between LD-Si and LD groups (*p* > 0.05).

The parameters obtained from the sum of BA contents were significantly altered in the LD group with respect to the ED group (*p* < 0.001) and recovered to some extent in the LD-Si group (*p* < 0.001) (Figure 1C–M). LD rats showed very significantly increases in all calculated BA amounts (ranged from 3.4- to 34.4- fold, *p* < 0.0001), except SBA and cSBA compared to ED rats. Silicon increased TBA, non-cBA, cBA, PBA, cPBA, 12α-OHBA contents and the ratios of non-cBA/cBA and 12α-OHBA/non-12α-OHBA (which ranged from 2.3- to 7.5-fold, *p* < 0.001–0.0001), without changes in SBA, cSBA and non-12α-OHBA (*p* > 0.05) compared to ED rats. Compared to LD animals, LD-Si rats showed significant decreases in TBA cBA, SBA cSBA, 12α-OHBA contents and non-cBA/cBA and 12α-OHBA/non-12α-OHBA ratios (which ranged from 2.64- to 3.94-fold, *p* < 0.001), while SBA content increased (2.80-fold, *p* = 0.003). No significant differences in non-cBA, cSBA and non-12α-OHBA contents between LD and LD-Si groups were found (*p* > 0.05).

To determine whether there is an association between the amounts of BA in the cecum and the severity of diabetic dyslipidemia, Pearson’s correlation coefficients (*p* < 0.05) were analyzed (Figure 1N). Significant differences have been marked with asterisks. We found that diabetes (DTscore) and dyslipidemia (DDscore) scores were positively correlated with the contents of CA, CDCA, α-MCA, HCA, GCA, TCA, TBA, PBA and the 12α-OHBA/non 12α-OHBA ratio (ranging from r = 0.417 to 0.921; *p* = 0.05 to 0.0001) and negatively correlated to DCA, LCA, oLCA, HDCA, TDCA and SBA contents (ranging from r = −0.722 to −0.570; *p* = 0.001–0.01).

### 2.4. Ileal BA Transporters and FXR and FGF15 Levels

**Figure 2:**
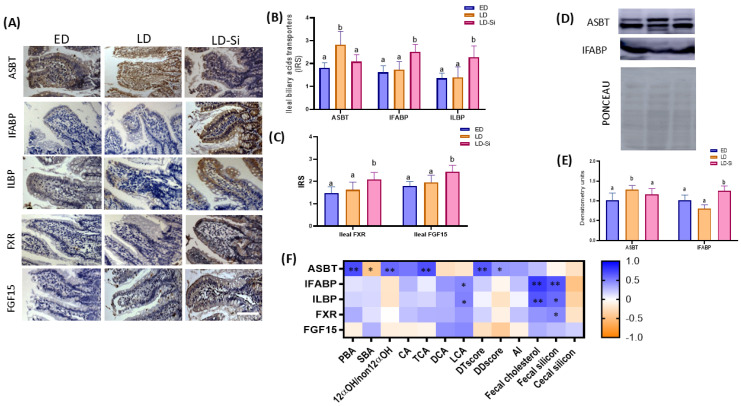
BA transporters and FXR and FGF15 levels of the ileal epithelium in the early-stage diabetes (ED), late-stage diabetes (LD) and Si-RM consumption in late-stage diabetes (LD-Si) groups. (**A**) Representative images of immunohistochemistry labeling of apical sodium-dependent bile acid transporter (ASBT), intestinal fatty acid binding protein (I-FABP) and ileal lipid binding protein (ILBP) transporters, farnesoid X receptor (FXR) and fibroblast growth factor 15 (FGF15). Scale bar: 50 µm. (**B**) Immunoreactivity score (IRS) of ASBT, IFABP and ILBP. (**C**) IRS of FXR and FGF15. (**D**) ASBT and IFABP bands of Western blot and Ponceau as loading control. (**E**) Percentage data of densitometric quantification for ileal ASBT (n = 7) and IFABP (n = 4). Values expressed as mean ± SD. Different letters (a < b) indicate significant differences between groups, Kruskal–Wallis and Dunn’s tests (*p* < 0.05). (**F**) Heatmap showing correlations between ASBT, IFABP, ILBP, FXR and FGF15 proteins with PBA, SBA, 12α-OHBA/non-12α-OHBA ratio, CA, TCA, DCA, LCA, DTscore, DDscore, atherogenic index (AI), fecal cholesterol, fecal daily silicon and cecal mucosa silicon contents. The color intensity of the heatmap represents the degree of association: Blue, positive association; Orange, negative. * Denotes adjusted: * *p* < 0.01, ** *p* < 0.001. Spearman’s correlation coefficient values were used for the matrix.

The immunolocalization and immunoreactivity scores (IRSs) of three transporters: the ileal apical sodium bile salt transporter (ASBT), intestinal fatty acid binding protein (I-FABP) and ileal lipid binding protein (ILBP), as well as the levels of ileal FXR and FGF15, are presented in Figure 2A–C (Kruskal–Wallis and Dunn’s tests, *p* < 0.05). ASBT and I-FABP levels were also assayed by Western blotting (Figure 2D,E) (ANOVA, Bonferroni post hoc test, *p* < 0.05). Figure 2F illustrates the correlations between ileal ASBT, I-FABP, ILBP, FXR and FGF15 levels and PBA, SBA, 12α-OHBA/non-12α-OHBA ratio, CA, TCA, DCA, LCA, DTscore, DDscore, AI, fecal cholesterol, fecal silicon and cecal silicon contents (Pearson’s or Spearman’s correlation coefficients (r), *p* < 0.05).

The LD group exhibited the highest ASBT levels, while no significant differences in IFABP and ILBP levels were observed with respect to the ED group (*p* > 0.05). The LD-Si group showed the highest levels of IFABP and ILBP protein transporters compared to the ED group (ranging from 21.4–66.1% (*p* = 0.0002)) and to the LD group (ranging from 34.9–151.3% (*p* < 0.0001)). No significant changes in FXR and FGF15 levels were observed in LD rats compared to ED rats (*p* > 0.05). However, Si-RM intake significantly increased the ileal levels of both FXR (35%, *p* < 0.0001) and FGF15 (28.2%, *p* < 0.0001) compared to LD rats. Compared to the ED group, Si-RM intake led to a 41.2% increase in FXR and a 23.9% increase in FGF15 levels (*p* < 0.001).

ASBT protein levels were positively associated with PBA (r = 0.708; *p* = 0.001), 12α-OHBA/non-12α-OHBA ratio (r = 0.589; *p* = 0·010), CA (r = 0.536; *p* = 0·022), TCA (r = 0.689; *p* = 0·0002), DTscore (r = 0.656, *p* = 0.0003) and DDscore (r = 0.550, *p* = 0.040), while they were negatively associated with SBA (r = −0.452: *p* = 0.049). Additionally, IFABP and ILBP transporter proteins were correlated positively with LCA (IFABP: r = 0.470, *p* = 0.040; ILBP: r = 0.488, *p* = 0.042), fecal cholesterol (IFABP: r = 0.771, *p* = 0.003; ILBP: r = 0.668, *p* = 0.018) and fecal silicon (IFABP: r = 0.674, *p* = 0.002; ILBP: r = 0.581, *p* = 0.010). Finally, intestinal FXR levels were correlated to fecal silicon (r = 0.463, *p* = 0.041) (Figure 2F).

### 2.5. Hepatic BA and Cholesterol Metabolism Markers

**Figure 3:**
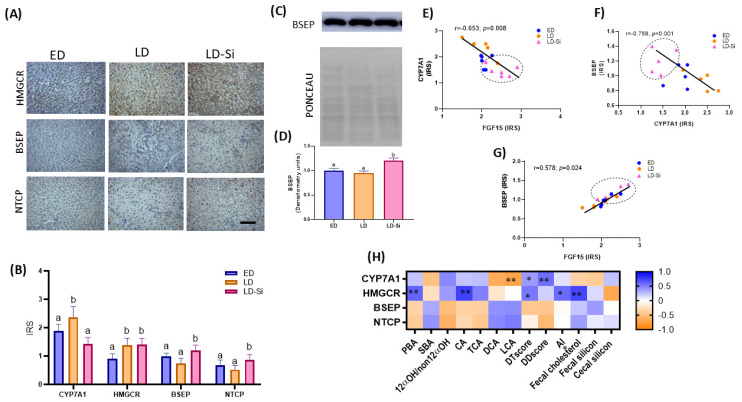
Hepatic CYP7A1, HMCGR, BSEP and NTCP levels in the early-stage diabetes (ED), late-stage diabetes (LD) and Si-RM consumption in late-stage diabetes (LD-Si) groups. (**A**) Representative images of immunohistochemistry labeling of cholesterol 7 alpha-hydroxylase1 (CYP7A1), 3-hydroxy-3-methylglutaryl CoA reductase (HMGCR), bile salt export pump (BSEP) and Na^+^-taurocholate co-transporting polypeptide (NTCP). Scale bar: 50 µm. (**B**) Immunoreactivity score (IRS) of CYP7A1, HMCGR, BSEP and NTCP. (**C**) BSEP and Ponceau (loading control) bands from Western blot. (**D**) Percentage data of densitometric quantification for BSEP. Values expressed as mean ± SD (n = 8). Different letters (a < b) indicate significant differences between groups, Kruskal–Wallis and Dunn’s tests (*p* < 0.05). Correlations showing linear regression line and Spearman’s coefficient and significance (*p* < 0.05) between (**E**) hepatic CYP7A1 and intestinal FGF15, (**F**) hepatic BSEP and CYP7A1, (**G**) hepatic BSEP and intestinal FGF15 levels. LD-Si group values are marked with a circle. (**H**) Heatmap showing correlations between CYP7A1, HMCGR, BSEP and NTCP proteins with PBA, SBA, 12α-OHBA/non-12α-OHBA ratio, CA, TCA, DCA, LCA, DTscore, DDscore, atherogenic index (AI), fecal cholesterol, fecal daily silicon and cecal mucosa silicon contents. The color intensity of the heatmap represents the degree of association: Blue, positive association; Orange, negative. * Denotes adjusted: * *p* < 0.01, ** *p* < 0.001. Spearman’s correlation coefficient values were used for the matrix.

Figure 3A,B show photographs representing the immunostaining and IRS of the hepatic CYP7A1 and 3-methylglutyl CoA reductase (HMGCR) enzymes and the BSEP and NTCP protein transporters (Kruskal–Wallis and Dunn’s tests, *p* < 0.05). BSEP levels were also measured by Western blotting (Figure 3C,D) (ANOVA, Bonferroni post hoc test, *p* < 0.05). Figure 3E–G show the correlations related to BA and cholesterol synthesis and uptake and efflux of BA and cholesterol in the liver (Pearson’s or Spearman’s correlation coefficients (r), *p* <0.05).

Compared to the ED group, CYP7A1 and HMGCR levels were significantly up-regulated (*p* < 0.05) in LD rats. However, consumption of Si-RM significantly increased CYP27A1 levels (*p* < 0.001) but did not modify the hepatic HMGCR levels (*p* > 0.05) compared to LD rats. Regarding BA transporters, liver photographs showed moderate diffuse staining of BSEP and NTCP protein levels distributed across the liver surface (Figure 3A). BSEP (IRS: 62.1%, *p* < 0.0001; WB: 22.8%, *p* < 0.001) and NTCP levels (IRS: 67.3%, *p* < 0.0001) were higher in the LD-Si group compared to the LD group (Figure 3B–D). No significant differences were found between ED and LD groups in these transporters.

We found a negative correlation between hepatic CYP7A1 and FGF15 levels (r = −0.653; *p* = 0.008) (Figure 3E) and between hepatic BSEP and CYP7A1 (r = −0.785, 0.01) (Figure 3F). However, the correlation between hepatic BSEP and intestinal FGF15 levels was positive, as shown in Figure 3G (r = 0.578; *p* = 0.024). In addition, inverse relationships were found between CYP7A1 and cecal DCA (r = −0.646, *p* = 0.009) and LCA contents (r = −0.714, *p* = 0.0004) (Figure 3H). HMGCR proportionally increased with respect to the levels of PBA, CA, DTscore, DDscore, AI and feces cholesterol (ranging from r = 0.500 to r = 0.704, *p* = 0.048–0.001) (Figure 3G).

### 2.6. Hepatic Cholesterol Efflux Markers

**Figure 4:**
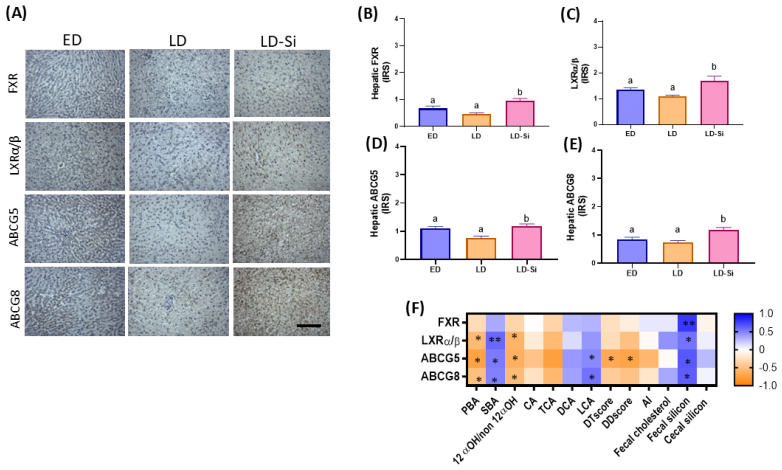
Hepatic FXR, LXRα/β, ABCG5 and ABCG8 in the early-stage diabetes (ED), late-stage diabetes (LD) and Si-RM consumption in late-stage diabetes (LD-Si) groups. (**A**) Representative images of immunohistochemistry labeling of hepatic farnesoid X receptor (FXR), liver X receptor transcription factor (LXRα/β), ATP-binding cassette subfamily G members 5 (ABCG5) and 8 (ABCG8). Scale bar: 50 µm. Immunoreactivity score (IRS) of hepatic (**B**) FXR, (**C**) LXRα/β, (**D**) ABCG5 and (**E**) ABCG8. Values expressed as mean ± SD. Different letters (a < b) indicate significant differences between groups, Kruskal–Wallis and Dunn’s tests (*p* < 0.05). (**F**) Heatmap showing correlations between hepatic FXR, LXRα/β, ABCG5, ABCG8 proteins with PBA, SBA, 12α-OHBA/non-12α-OHBA ratio, CA, TCA, DCA, LCA, DTscore, DDscore, atherogenic index (AI), fecal cholesterol and fecal silicon contents and cecal mucosa silicon content. The color intensity of the heatmap represents the degree of association: Blue, positive association; Orange, negative. * Denotes adjusted: * *p* < 0.01, ** *p* < 0.001. Spearman’s correlation coefficients values were used for the matrix.

Figure 4 illustrates photographs representing the immunostaining (Figure 4A) and IRS of hepatic proteins FXR, LXRα/β, ABCG5 and ABCG8 (Figure 4B–E) (Kruskal–Wallis and Dunn’s tests, *p* < 0.05). Figure 4F shows the correlations related to efflux cholesterol markers in the liver (Spearman’s correlation coefficients (r), *p* < 0.05).

The LD group showed a tendency towards a decrease in hepatic FXR, LXRα/β ABCG5 and ABCG8 levels, although no significant differences were found with respect to the ED group. LD-Si rats showed higher levels of these proteins compared to their LD counterparts (FXR: 60.0% (*p* < 0.001); LXRα/β: 59.4% (*p* = 0.001); ABCG5: 55.2% (*p* = 0.033); ABCG8: 61.6% (*p* = 0.01)), showing levels similar to those of the ED animals (*p* > 0.05).

LXRα/β and ABCG5/8 levels were positively correlated to PBA and 12α-OHBA/non-12α-OHBA ratio and negatively correlated to SBA and LCA levels (*p* < 0.01 to *p* < 0.0001). FXR, LXRα/β and ABCG5/8 levels were dependent on daily fecal silicon content (*p* < 0.05) (Figure 4F).

### 2.7. Ileal Cholesterol Absorption and Dietary and Transintestinal Cholesterol Efflux (TICE) Markers

**Figure 5:**
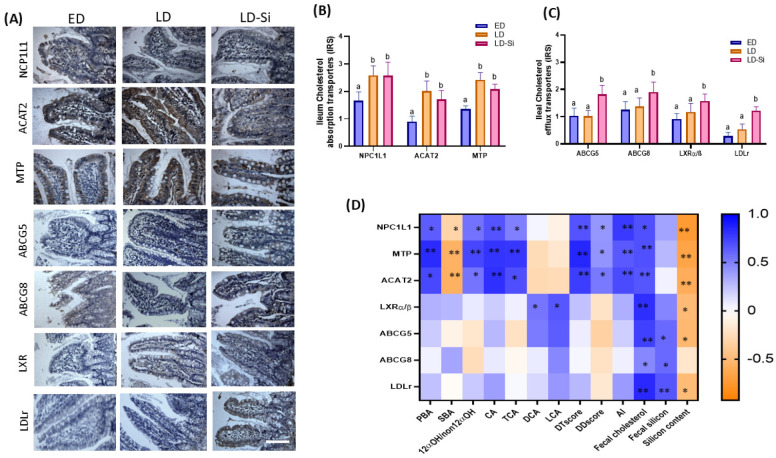
Ileal Niemann–Pick C1-Like 1 (NPC1L1), acetyl-coenzyme A acetyltransferase-2 (ACAT2),microsomal triglyceride transfer protein (MTP), liver X receptor transcription factor (LXRα/β), ATP-binding cassette subfamily G members 5 (ABCG5) and 8 (ABCG8) and low-density lipoprotein receptor (LDLr) in the early-stage diabetes (ED), late-stage diabetes (LD) and Si-RM consumption in late-stage diabetes (LD-Si) groups. (**A**) Representative images of immunohistochemistry labeling of ileal NCP1L1, ACAT2, MTP, LXRα/β, ABCG5, ABCG8 and LDLr. Scale bar: 50 µm. (**B**) Immunoreactivity score (IRS) of ileal NCP1L1, ACAT2 and MTP. (**C**) IRS of ileal LXRα/β, ABCG5, ABCG8 and LDLr. Values expressed as mean ± SD. Different letters (a < b) indicate significant differences between groups, Kruskal–Wallis and Dunn’s tests (*p* < 0.05). (**D**) Heatmap showing correlations between NCP1L1, ACAT2, MTP, LXRα/β, ABCG5, ABCG8 and LDLr proteins with PBA, SBA, 12α-OHBA/non-12α-OHBA ratio, CA, TCA, DCA, LCA, DTscore, DDscore, atherogenic index (AI), fecal cholesterol, fecal daily silicon and cecal mucosa silicon contents. The color intensity of the heatmap represents the degree of association: Blue, positive association; Orange, negative. * Denotes adjusted: * *p* < 0.01, ** *p* < 0.001. Pearson’s or Spearman’s correlation coefficient values were used for the matrix.

The levels of ileal cholesterol absorption and efflux transporters are shown in Figure 5. The NPC1L1, ACAT2 and MTP levels were measured by immunohistochemistry (Figure 5A,B). Figure 5C shows the immunohistochemical detection of ileal ABCG5, ABCG8, LXRα/β and LDLr proteins (Kruskal–Wallis and Dunn’s tests, *p* < 0.05). The results from Sperman correlations related to ileal cholesterol absorption and efflux are shown in Figure 5D (Spearman’s correlation coefficients (r), *p* < 0.05).

Moderate to strong immunoreactivity intensities were found in all groups, as shown in Figure 5A. The LD group displayed higher NPC1L1 (56.0%, *p* = 0.0009), ACAT2 (123.3%, *p* < 0.00001) and MTP (77.9%, *p* = 0.008) transporter levels than the ED group (Figure 5B). However, no significant differences were observed in the IRS of ABCG5, ABCG8, LXRα/β and LDLr proteins between LD and ED groups (*p* > 0.05) (Figure 5C). Regarding silicon’s effect, no differences in cholesterol absorption transporters between the LD and LD-Si groups were found (*p* > 0.05). LD-Si rats showed higher levels of ABCG5 (76.6%, *p* < 0.001), ABCG8 (52.3%, *p* < 0.001), LXRα/β (72.5%, *p* < 0.001) and LDLr (505.0%, *p* = 0.0002) than ED rats and also compared to their LD counterparts (ABCG5: 78.4%, *p* < 0.001, ABCG8: 38.4%, *p* < 0.001, LXRα/β: 33.0%, *p* = 0.0001 and LDLr: 128.3%, *p* < 0.0001).

NCP1L1, MTP, and ACAT2 levels were directly correlated with PBA, 12α-OHBA/non-12α-OHBA ratio, CA, TCA, DTscore, DDscore and AI (ranging from r = 0.551 to 0.888, *p* = 0.018–0.002) and inversely correlated with SBA (range r = −0.500 to −0.571, *p* = 0.018–0.001). No significant correlations between FXR, LXRα/β, ABCG5/8 and LDLr levels and BA pools and glycolipid metabolism parameters were found (*p* > 0.05). Additionally, cholesterol feces content was positively correlated with all the studied ileal cholesterol absorption and efflux parameters (*p* < 0.046, *p* < 0.0001), while ABCG5/8 and LDLr levels were positively correlated with fecal silicon content. NCP1L1, MTP, ACAT2, LXRα/β, ABCG5 and LDLr levels were inversely proportional to silicon content of the cecal mucosa (*p* < 0.05) (Figure 5D).

### 2.8. Ileal Morphometric Parameters and Integrity Barrier

**Figure 6:**
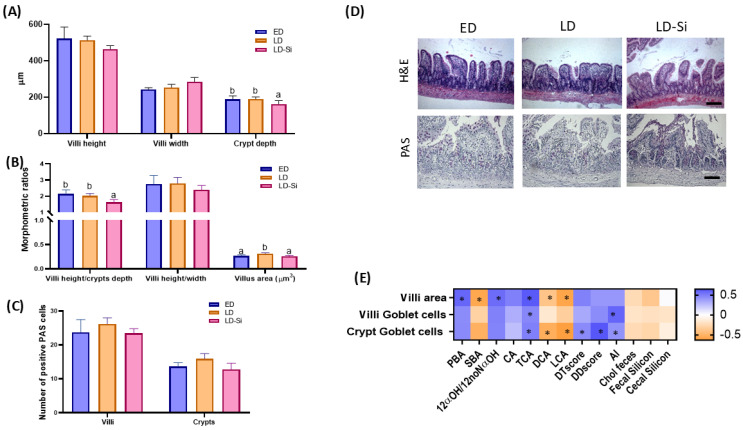
Morphometric parameter and number of goblet cells of the ileal epithelium in early-stage diabetes (ED), late-stage diabetes (LD) and Si-RM consumption in late-stage diabetes (LD-Si) groups. (**A**) Villi height, width and crypt depth (µm). (**B**) Villi height/width and villi height/crypt ratios and villi area (µm^3^). (**C**) Number of goblets cells of villi and crypts from periodic acid–Schiff staining (PAS) slices. Values expressed as mean ± SD. Different letters (a < b) indicate significant differences between groups, ANOVA, Bonferroni post hoc test (*p* < 0.05). (**D**) Representative images of histochemistry by hematoxylin and eosin (H&E), scale bar: 100 µm and PAS staining, scale bar: 50 µm. (**E**) Heatmap showing correlations between villi area, number of villi goblet cells and crypt goblet cells with PBA, SBA, 12α-OHBA/non-12α-OHBA ratio, CA, TCA, DCA, LCA, DTscore, DDscore, atherogenic index (AI), fecal cholesterol, fecal daily silicon and cecal mucosa silicon contents. The color intensity of the heatmap represents the association degree: Blue, positive association; Orange, negative. * Denotes adjusted: * *p* < 0.01. Pearson’s correlation coefficient values were used for the matrix.

Ileal mucosae morphologic parameters from H&E staining and the number of positive periodic acid–Schiff stain (PAS) cells are displayed in Figure 6A–D (ANOVA, Bonferroni post hoc test, *p* < 0.05). Changes in ileal mucosa morphology and villi and crypt numbers of PAS cells related to PBA, SBA, 12α-OHBA/non-12α-OHBA ratio, CA, TCA, DCA, LCA, DTscore, DDscore, AI and cholesterol feces parameters are shown in Figure 6E (Pearson’s correlation coefficients (r), *p* < 0.05).

Figure 6A–D shows the morphologic changes in the ileal mucosae. The villi height, the villi width and the ratios of villi height/crypt depth and villi height/width, along with the number of PAS villi and crypt goblets, showed no significant differences between the three groups (*p* > 0.05). However, Si-RM consumption significantly decreased the absorptive area (−16.4%, *p* = 0.004) compared to LD animals, reaching the values of ED rats (*p* > 0.05) (Figure 6C). Moreover, LD-Si rats showed significantly deeper crypts than ED (−14.2%, *p* = 0.023) and LD rats (−15.4%, *p* = 0.021) (Figure 6A), leading to a lower villi height/crypt depth ratio (*p* < 0.05) (Figure 6B). As indicated by the significant Pearson’s coefficients, the villi area was positively affected by PBA, 12α-OHBA/non-12α-OHBA ratio and TCA (ranged values, r = 0.632 to r = 0.564, *p* = 0.001–0.026) and negatively by SBA, DCA and LCA (ranged values, r = −0.596 to r = −0.397, *p* = 0.006–0.020). The number of PAS-positive cells in villi was directly proportional to AI (r = 0.590, *p* = 0.01) and TCA (r = 0.457, *p* = 0.02), while PAS cells in crypts were positively affected by TBA, DTscore, DDscore and AI (ranged values, r = 0.645 to r = 0.416, *p* = 0.001–0.02) and negatively by DCA and LCA (ranged values, r = −0.632 to r = −0.542, *p* = 0.006–0.001).

**Figure 7:**
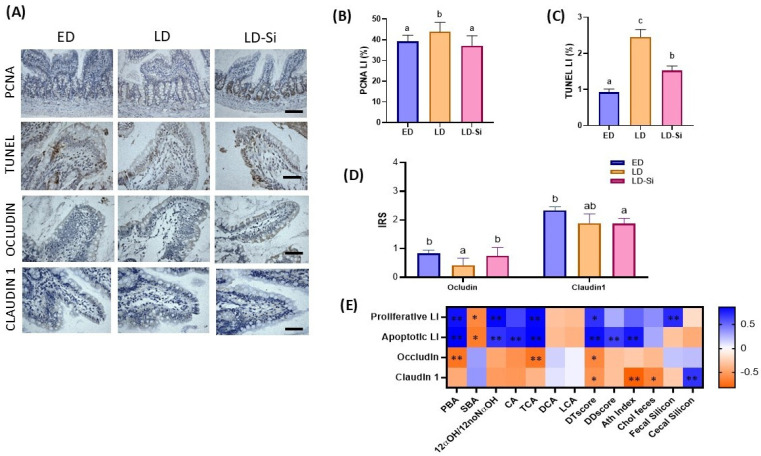
Proliferation and apoptosis indexes and tight junctions’ barrier integrity of the ileal epithelium in early-stage diabetes (ED), late-stage diabetes (LD) and Si-RM consumption in late-stage diabetes (LD-Si) groups. (**A**) Representative images of immunohistochemistry marking proliferating cell nuclear antigen (PCNA), scale bar: 250 µm, ileal apoptotic positive nuclei by terminal deoxynucleotidyl transferase dUTP nick end labeling (TUNEL), scale bar: 20 µm and claudin-1 and occludin levels, scale bar: 20 µm. (**B**) Immunoreactivity score (IRS) of crypt PCNA labeling index (PCNA-LI) (%), (**C**) Positive villi apoptotic nuclei from TUNEL labeling index (TUNEL-LI) (%). (**D**) IRS of occludin and claudin-1. Values expressed as mean ± SD. Different letters (a < b < c) indicate significant differences between groups, ANOVA, Bonferroni post hoc test or Kruskal–Wallis and Dunn’s tests (*p* < 0.05). (**E**) Heatmap showing correlations between PCNA-LI, TUNEL-LI, claudin-1 and occludin with PBA, SBA, 12α-OHBA/non-12α-OHBA ratio, CA, TCA, DCA, LCA, DTscore, DDscore, atherogenic index (AI), fecal cholesterol, fecal daily silicon and cecal mucosa silicon contents. The color intensity of the heatmap represents the degree of association: Blue, positive association; Orange, negative. * Denotes adjusted: * *p* < 0.01, ** *p* < 0.001. Pearson’s or Spearman’s correlation coefficient values were used for the matrix.

The proliferation and apoptosis balance in the intestinal epithelium (ANOVA, Bonferroni post hoc test, *p* < 0.05) and the levels of claudin-1 and occludin tight junction proteins (Kruskal–Wallis and Dunn’s tests, *p* < 0.05) are displayed in Figure 7A–D. Photographs of the PCNA, TUNEL and tight junction immunohistochemistry are shown in Figure 7A. Changes in ileal turnover and the integrity of the intestinal barrier related to PBA, SBA, 12α-OHBA/non-12α-OHBA ratio, CA, TCA, DCA, LCA, DTscore, DDscore, AI and cholesterol feces parameters are shown in Figure 7E (Pearson’s or Spearman’s correlation coefficients (r), *p* < 0.05).

Intestinal proliferation and apoptosis were measured by PCNA labeling index (PCNA-LI) (Figure 7B) and TUNEL labeling index (TUNEL-LI) (Figure 7C), respectively. The LD group showed increased epithelial turnover in the ileal mucosa, with the highest PCNA-LI in the crypts (15.3%, *p* = 0.01) and TUNEL-LI in the villi (169.2%, *p* < 0.0001) with respect to the ED group. The LD-Si group displayed lower levels in both PCNA-LI (15.6%, *p* = 0.01) and TUNEL-LI (37.9%, *p* = 0.004) compared to LD rats, but only reached ED values for PCNA-LI, maintaining a higher TUNEL-LI signal. As indicated by the significant Pearson coefficients, PCNA-LI and TUNEL-LI were positively affected by PBA, 12α-OHBA/non-12α-OHBA ratio TCA and DTscore (ranged values, r = 0.852 to r = 0.636, *p* = 0.001–0.026) and negatively by SBA (PCNA-LI r = −0.616, TUNEL-LI r = −0.677, *p* = 0.006–0.020). Additionally, PCNA-LI was positively correlated to fecal silicon content and negatively correlated to silicon content in the cecum (*p* = 0.001).

Figure 7C shows the IRS of occludin and claudin-1 of the ileal mucosae. We observed a differential pattern in the immunoreactivity of the tight junction proteins. The ileal absorptive barrier was altered in the late-stage T2DM rats, as shown by the lower occludin (*p* = 0.002) and claudin-1 (*p* = 0.046) levels in the LD group compared to the ED group. Si-RM consumption significantly increased occludin levels compared to LD animals, but they were similar to those of the ED group (*p* > 0.05). Regarding claudin-1 levels, the LD-Si group had similar levels to those of its LD counterpart and lower levels than ED animals (*p* = 0.01). There was a negative correlation between occludin levels and PBA (r = −0.694, *p* = 0.00) and TCA (r = −0.642, *p* = 0.004) contents. Claudin-1 and occludin were significantly correlated with DTscore (*p* > 0.001), while claudin-1 was correlated with AI (*p* > 0.001), fecal cholesterol (*p* = 0.01) and cecal mucosae silicon content (*p* = 0.001) (Figure 7D).

### 2.9. Cecal Short-Chain Fatty Acid Composition

**Figure 8:**
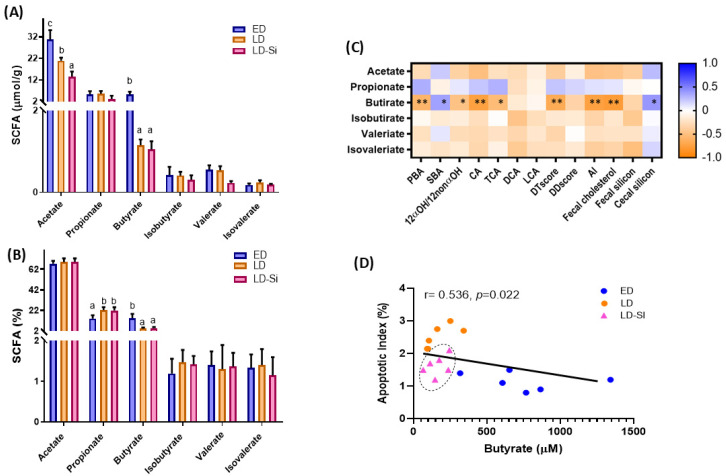
Short chain fatty acid (SCFA) content in cecum of early-stage diabetes (ED), late-stage diabetes (LD) and Si-RM consumption in late-stage diabetes (LD-Si) groups. (**A**) SCFA profile (µmol/g). (**B**) Percentage of SCFA. Values expressed as mean ± SD. Different letters (a < b < c) indicate significant differences between groups, ANOVA, Bonferroni post hoc test (*p* < 0.05). (**C**) Heatmap showing correlations between acetate, propionate, butyrate, isobutyrate, valerate and isovalerate with PBA, SBA, 12α-OHBA/non-12α-OHBA ratio, CA, TCA, DCA, LCA, DTscore, DDscore, atherogenic index (AI), fecal cholesterol, fecal daily silicon and cecal mucosa silicon contents. The color intensity of the heatmap represents the degree of association: Blue, positive association; Orange, negative. * Denotes adjusted: * *p* < 0.01, ** *p* < 0.001. Pearson’s correlation coefficient values were used for the matrix. (**D**) Pearson correlation between apoptotic index as TUNEL-LI (%) and butyrate content (µmol/g) showing Pearson’s coefficients and significance (*p* < 0.05). LD-Si group values are marked in the circle.

Cecal SCFA content and percentage are presented in Figure 8A,B (ANOVA, Bonferroni post hoc test, *p* < 0.05). Figure 8C,D show the correlations relative to cecal SCFA (Pearson’s correlation coefficients (r), *p* < 0.05).

The levels of acetate and butyrate were lower in LD rats than in the ED rats (Figure 8A; *p* < 0.05). No difference was found between LD and ED groups in propionate, isobutyrate, valerate and isovalerate contents (*p* > 0.05). However, the percentage of propionate was higher, while butyrate was lower in LD rats compared to ED rats (Figure 8B). Si-RM intake had little effect on SCFA levels (*p* > 0.05), although it notably decreased the cecal acetate content compared to both LD (56.1%, *p* = 0.001) and ED (35.13%, *p* = 0.01) groups (Figure 8A). In addition, only butyrate was positively correlated with SBA (r = 0.680, r = 0.001) and cecal mucosa silicon content and negatively correlated to PBA, 12α-OHBA/non-12α-OHBA ratio, CA, TCA, DTscore, AI and fecal cholesterol content (*p* < 0.03) (Figure 8C). A negative correlation between apoptotic index (TUNEL-LI) and butyrate level was found (r = 0.536, *p* = 0.022) (Figure 8D).

## 3. Discussion

Previous studies have shown that a long-term consumption of silicon incorporated into a meat matrix (Si-RM) has a cholesterol-lowering effect, improving hyperglycemia and hyperlipidemia in metabolic syndrome and T2DM rat models [33,34,35,36]. In this study, we explored the potential effects of Si-RM consumption on BA metabolic processes and their underlying mechanisms, which could be partly responsible for its hypocholesterolemic effect. The impact of silicon on cecal BA and SCFA, metabolites from intestinal microbiota, as well as the integrity of the intestinal barrier, has been studied. The most important findings were that silicon: (1) reduced the total cecal BA amount and improved the hydrophilic–hydrophobic balance of BAs that escape from the enterohepatic circulation by inhibiting ileal ASBT protein transporter; (2) prevented the hepatic BA synthesis through up-regulation of the FXR/FGF15/CYP7A1 pathway in the gut–liver axis; (3) up-regulated hepatic and ileal FXR and LXRα/β levels, facilitating biliary and transintestinal cholesterol efflux (TICE) by the activation of ABCG5/8 transporters; (4) improved the integrity of the ileal barrier by an up-regulation of tight junctions and promoting a less intestinal toxic environment. These results confirmed that the hypoglycemic and hypocholesterolemic effects of silicon, as a functional ingredient included in a meat matrix, could be an effective nutritional adjuvant in therapeutic strategies for the treatment of diabetic dyslipidemia.

The results of the current study supported that the progression from early- to late-stage T2DM (LD vs. ED) entails changes in the cecal content and profile of BA. Quantitative analysis of cecal BA content confirmed that LD rats had higher amounts of PBA, including TCA, CA, αMCA, CDCA and HCA, and lower SBA contents, such as oLCA and HDCA, than their ED counterparts, while there were no changes in CDA and LCA levels. This BA profile, rich in PBA with respect to SBA, differs from that found in the cecum of healthy rats, where unabsorbed PBAs are fully modified into SBAs by the gut microbiota in the large intestine, mainly in the ileum and cecum, before their colonic absorption or fecal excretion [11,13]. An altered BA amount and composition can significantly impact gastrointestinal homeostasis, liver function and overall metabolic balance, inducing, in turn, insulin resistance and diabetic dyslipidemia [7]. Specifically, TCA and CDA contents were positively associated with the intake of atherogenic diet, increasing the risk of T2DM development in humans [17]. Furthermore, a higher proportion of conjugated versus unconjugated BA is linked to alterations in hepatic catabolism and/or intestinal reabsorption of BA, contributing to the development of TD2M [38]. As a consequence of anomalous BA profile, the 12α-OHBA/non-12α-OHBA ratio was increased in the LD rats. In agreement with our results, a higher 12α-OHBA/non-12α-OHBA ratio had been found in different diabetic rat models [39,40,41]. The increases in 12α-OHBA and cBA, which are more effective than non-12α-OHBA and non-cBA at emulsifying dietary fat [42], might promote better digestion and absorption of fat in LD rats, contributing to hypercholesterolemia. Specifically, increased levels of CA and TCA contributed to diabetic dyslipidemia by stimulating cholesterol absorption [43]. Cecal BA results were significantly correlated to DTscore and DDscore, revealing the relationship between the altered BA contents and the development of diabetic dyslipidemia.

Si-RM consumption improved the cecal BA amounts and composition in comparison to LD control, bringing them closer to those of the ED rats. Cecal TBA, cBA/non-cBA, PBA and 12α-OHBA/non-12α-OHBA ratio decreased, while SBA increased in LD-Si rats with respect to LD rats, shifting the BA pool toward a more hydrophilic profile with a lower capacity to digest dietary fat [40]. Although the BA profile was not significantly modified by silicon, LD-Si rats showed lower CA and TCA contents and higher amounts of DCA, LCA and oLCA with respect to LD rats, achieving BA levels of ED rats. The decrease in hydrophobic BA could prevent cholesterol absorption by the diminution of intraluminal micellar cholesterol solubilization [44], increasing cholesterol fecal excretion. This mechanism could reinforce another that we previously hypothesized including the capacity of silicon to create molecular crosslinks in the intestinal lumen, allowing the formation of insoluble and non-absorbable esters by interaction with the micellar formation [33,34,35,36], preventing fat absorption. In this sense, silicon could act as a BA sequestrant, removing BA from the enterohepatic circulation. It is known that BA sequestrants increased the return of LDLc to the liver [45] and also improved glycemic control in patients with T2DM [46].

To explore the mechanism underlying BA excretion induced by Si-RM consumption, we next analyzed the ileal and hepatic levels of proteins related to BA and cholesterol metabolisms. BA concentration, composition and circulation are tightly regulated by a complex system of coordinated membrane transporters and biosynthetic enzymes. Ileal luminal ASBT and cytoplasmatic I-FABP and ILBP transporters promote BA absorption and their enterohepatic recycling [47,48]. LD rats showed higher levels of ASBT but no significant differences in I-FABP and ILBP levels compared to ED rats. Although up-regulation of BA transporters depends on the activation of FXR [49], in our study, no changes in ileal FXR levels were found in LD respect to ED rats. The enlargement of luminal BA might induce up-regulation of ASBTs as occurred in T2DM [50]. Furthermore, the excess of dietary cholesterol and CA (HSFHCD) consumed by LD rats could be responsible for altering the ileal BA absorption mechanism. According to our results, the supplementation of CA in LD animals, differently to their ED counterparts, could up-regulate ASBT expression, activating enterohepatic circulation for potentially protecting the organism from the toxic effects of excessive SBA production in the colon [51]. The absence of changes in FXR levels could be partially attributed to BA percentages, finding TCA, which is a weak FXR agonist, as the main cecal BA in LD rats (80.33%) and strongly increased with respect to the ED group (28.49%), while CDCA, with the greatest FXR activation potential [52], showed a very poor contribution in the BA pool of LD animals (0.07%).

The effects of the Si-RM consumption also targeted ileum BA transporters. We observed a significant decrease in ASBT levels in LD-Si rats with respect to LD rats, accompanied by an increase in IFABP and ILBP levels and FXR activation, which could potentially be induced by a lower luminal percentage of TCA (53.81%). The reduction in ASBT could have played an adaptive role by adjusting the enterohepatic circulation to the restricted flow of BA in the ileum, as discussed below with CYP7A1 results. The down-regulation of ASBT by silicon depended on the luminal content of PBA, specifically CA and TCA, and the 12α-OHBA/non-12α-OHBA ratio as indicated by the significant and positive correlations found. Furthermore, ASBT levels showed significant correlations with DTscores and DDscores, suggesting a double benefit by controlling hyperglycemia and hypercholesterolemia. It has been demonstrated that the down-regulation of ASBT is linked to a reduction in LDLc levels [53], explaining its systemic consequences. Like metformin [53], the most recognized drug in the treatment of T2DM, silicon reduced ASBT-mediated BA reabsorption in the terminal ileum and could be a new nutritional tool for the treatment of diabetic dyslipidemia.

Intestinal and hepatic FXR work together through the FXR/FGF19-15/CYP7A1 pathway in the gut–liver axis to establish a dynamic balance between synthesis and excretion in the BA pool size [54]. Intestinal FXR activation induces FGF15 expression in rodents, which is considered a major mechanism in suppressing hepatic BA synthesis through the inhibition of CYP7A1, the rate-limiting enzyme of BA synthesis and cholesterol catabolism in hepatocytes [55,56]. In fact, hepatic BA synthesis, particularly CA, is known to be increased in patients with T2DM and in rats fed a cholesterol-enriched diet, as a consequence of the dysregulation of the CYP7A1/FGF19/15 negative feedback pathway [56]. In our study, the uptake of BAs into intestinal cells and their reabsorption into the portal vein were abnormal in LD rats. No changes in levels of ileal FXR and FGF15 were found (*p* > 0.05), but hepatic CYP7A1 protein levels were not inhibited, as higher levels were observed compared to ED rats. However, although cholesterol elimination via BA excretion was activated in the LD group, HMGCR levels were also higher in LD rats compared to ED rats, contributing to increased endogenous cholesterol synthesis and hypercholesterolemia [57]. HMGCR overexpression also promoted insulin resistance, inflammatory response and hepatic steatosis in diet-induced obese mice [58]. Additionally, dysregulation of hepatic biliary uptake and excretion of BA was found in LD rats. Thus, non-significant differences in hepatic FXR, LXRα/β, NTCP, BSEP and ABCG5/8 levels were found between LD and ED rats (*p* > 0.05), despite the high cholesterol and CA intake in the LD group, leading to pathological cholesterol accumulation in the liver of LD rats [59,60]. The excess hepatic BA, due to both increased synthesis and enterohepatic circulation, could induce significant liver injury through intracellular BA accumulation and hepatotoxicity in our late-stage T2DM rats. In line with this, a previous study showed that LD rats had panlobular steatosis with macrovesicular and microvesicular intracellular lipid droplets (NAS, 7.25 ± 0.89) [61], likely as a result of the altered cholesterol and BA metabolism revealed in this work. Further studies are needed to assess the degree of hepatic damage caused by BA and cholesterol accumulation in the liver.

The effect of silicon also targeted the FXR/FGF15/CYP7A1 pathway. Si-RM intake activated the ileal FXR/FGF15 pathway, promoting the reduction of hepatic CYP7A1 levels, thereby decreasing the conversion of cholesterol into BA and consequently reducing the cecal BA pool size. An inverse correlation between hepatic CYP7A1 and intestinal FGF15 levels (r = −0.653; *p* = 0.008) confirmed the negative feedback between these parameters, leading to inhibition of BA synthesis. In addition, both parameters were dependent on DCA and LCA levels. These findings suggest that silicon might act as an intestinal-based FXR agonist capable of triggering substantial metabolic benefits [62]. Although, in our study, activation of both ileal and hepatic FXR did not correlate with DTscore and DDscore, multiple studies have shown that up-regulation of FXR is beneficial to improve T2DM, increasing hepatic glycogen synthesis and significantly improving hyperglycemia and hyperlipidemia [47,52,63,64]. Moreover, silicon maintained elevated levels of the liver enzyme HMGCR, but the excess of endogenous cholesterol could be secreted into bile via up-regulation of ABCG5/8 or directly from plasma into the intestinal lumen via TICE, as discussed below. With respect to biliary cholesterol efflux, silicon compensated for the increased cholesterol synthesis by up-regulating hepatic LXRα/β and FXR levels, which in turn promoted higher levels of BSEP and ABCG5/G8, responsible for the efflux of BA and cholesterol into bile canaliculi, respectively [63]. The synthesis of hepatic ABCG5/G8 through the activation of both LXRα/β and FXR was confirmed by the significantly positive correlations found between them (*p* < 0.03); while the up-regulation of BSEP depended on intestinal FGF15 levels and was negatively affected by the CYPT7A1 levels. On the other hand, the NTCP levels did not change between experimental groups (*p* > 0.05) and, therefore, the hepatic uptake of BA was not modified by the Si-RM consumption, maintaining the clearance of BA from the portal vein. In short, the positive regulation of hepatic FXR induced by silicon increased canalicular secretion by BSEP without altering sinusoidal uptake by NTCP, which could activate enterohepatic circulation. Additionally, the positive regulation of hepatic LXRα/β induced ABCG5/G8. Together, these mechanisms could prevent the accumulation of BA and cholesterol in the liver of LD-Si rats [65].

Next, we studied the hypocholesterolemic effect of silicon on ileal cholesterol abortion and efflux. LD rats had increased NPC1L1, ACAT2 and MTP protein levels, whereas no changes were observed in the LXRα/β-ABCG5/G8 pathway, favoring the cholesterol absorption but not its excretion. In addition, LD rats showed similar levels of ileal LDLr compared to ED rats, despite their greater cholesterol intake, which could compromise TICE, preventing the clearance of atherogenic lipoproteins from the basolateral membrane of enterocytes and the excretion of cholesterol through the apical transporters ABCG5/G8 [66]. Despite the increased cholesterol absorption, LD rats eliminated greater amounts of total fat and cholesterol in their feces compared to ED rats, suggesting that the supplemented dietary cholesterol exceeded the small intestine’s absorptive capacity and, consequently, “overflowed”, causing steatorrhea [36]. In contrast, although the cholesterol absorption transporters in ileal enterocytes were not modified by Si-RM consumption (LD-Si animals vs. LD ones), ileal cholesterol excretion was increased. Silicon’s activation of the luminal cholesterol efflux and TICE in the ileum, as also observed in the duodenum and jejunum of these rats [33,36], led to the largest fecal fat and cholesterol excretion compared to ED and LD groups. Furthermore, we found a synergistic activation of the LXRα/β and FXR on ABCG5/G8 as indicated by positive correlations found between them. Similarly, in this sense, the activation of the direct FXR target, enterokine FGF15/19, has been shown to stimulate robust secretion of cholesterol into the intestinal lumen via ABCG5/G8 and TICE in mice [67,68]. We also investigated whether the activation of cholesterol efflux after Si-RM consumption was related to the luminal fecal silicon content or to the silicon content in the intestinal mucosa. We found that the increase in LDLr and ABCG5/G8, which led to the activation of TICE and dietary cholesterol efflux, was dependent on luminal fecal silicon content, as demonstrated by positive correlations between daily fecal silicon levels and LXRα/β, ABCG5 and LDLr levels. These results indicate that silicon may not only act by sequestering fat in micelles within the lumen but also by positively up-regulating cholesterol efflux transporters, thereby promoting its excretion. Interestingly, an inverse correlation between silicon content in ileal mucosa and the absorption and efflux transporters was found. The silicon content in ileal mucosa was lower in LD and LD-Si rats than in their ED counterparts, but further studies are requested to determine whether this is a pathological marker of late-stage T2DM linked to a severe alteration of the lamina propria (normally rich in this mineral).

Finally, we examined the impact of the microbiota-derived metabolites induced by Si-RM consumption on intestinal barrier integrity. As biological detergents, hydrophobic BAs are inherently cytotoxic and a potential source of inflammatory stress in the liver or intestines, leading to DNA damage, cell death and altered intestinal barrier integrity [69] and permeability [70]. These alterations are particularly significant in the ileal compartment, as the conversion of PBA to SBA and the formation of SCFA from microbial metabolism begin here and continue throughout the large intestine. An imbalance of gut microbiota or their metabolites can cause alterations in the intestinal barrier integrity, which seems to be at the root of the low-grade inflammation characteristic of chronic diseases such as T2DM [70]. In this context, an increase in pathogenic bacteria, such as *Enterobacteriaceae*, various *Clostridiales, Escherichia coli*, *Bacteroides caccae*, and *Lactobacilli*, as well as *Prevotella copri* and *Bacteroides vulgates*, has been observed in the microbiota of diabetic patients [71]. LD rats exhibited a greater absorption area, lower expression of occludin and claudin-1 tight junctions and accelerated cell turnover with higher rates of cell proliferation and apoptosis in the ileum compared to ED rats. These ileal barrier alterations were also found in the mucosa of the duodenum and jejunum, where they promote fat absorption [33,36]. The positive correlations between TBA, PBA, hydrophobic 12α-OHBA, CA and TCA with PCNA-LI and TUNEL-LI indexes, and the inverse correlation with occludin (*p* > 0.01), demonstrated the involvement of BA in the damage of the ileal barrier in LD rats. Furthermore, we have previously reported that LD rats showed colonic homeostasis disruption, with dysbiosis and intestinal barrier dysfunction [31], which could facilitate the translocation of luminal toxins into the host [72]. Specifically, ileum epithelial cells in LD rats were continuously exposed to high levels of TCA, which may trigger barrier dysfunction. Consistent with our findings, a high proportion of TCA has been found in animals fed a Western diet [16,17] and in subjects consuming a diet rich in meat and saturated fats, causing a high genotoxic potential [72]. Moreover, taurine produced by bacterial deconjugation of TCA is used by *B. wadsworthia* as a substrate for anaerobic respiration, generating genotoxic H_2_S [73], which promotes colonocyte turnover, inflammatory bowel disease and colon cancer [74]. In addition, SCFAs are also products of microbiota metabolism and play an important role in maintaining intestinal barrier function [75]. SCFAs have been shown to increase insulin sensitivity and promote glucose homeostasis, so modulation of SCFA by silicon could provide a unique approach to managing T2DM [20]. Moreover, changes in SCFA composition and function may influence the concentrations of BA. In our study, the SCFAs associated with carbohydrate metabolism (acetate and butyrate) were significantly reduced in LD rats, while propionate, valerate, isovalerate and isobutyrate contents did not change with respect to ED rats. Patients with T2DM show a moderate degree of intestinal dysbiosis with a reduced abundance of butyrate-producing bacteria [76]. Furthermore, a reduction in SCFA led to a decrease in tight junction levels, resulting in the impairment of intestinal epithelial barrier integrity in LD rats [77]. Thus, the relationship between diet, hydrophobic BA contents and the final metabolic products of TCA metabolism, along with reduced acetate and butyrate content by intestinal bacteria, could alter the intestinal permeability and serve as potential precursors of T2DM progression in LD rats.

Silicon was able to dilute toxic BAs and make them less harmful to the host. The shift towards a hydrophilic profile of BA induced by Si-RM consumption triggered an adaptive response in the ileal mucosa, with a smaller absorption area and decreased epithelial cell turnover, which were dependent on the cecal SBA content and both cecal mucosa and feces silicon contents. Inverse associations between SBA content and the absorptive area, PCNA-LI and TUNEL-LI (*p* < 0.01) and between LCA content with the absorptive area and the number of ileal goblet cells (*p* < 0.001) were found. PCNA-LI positively correlated with fecal silicon content and negatively correlated to cecal mucosa silicon content, indicating its impact on the epithelial turnover. Additionally, the significant increase in occludin levels in LD-Si rats could help reinforce the intestinal barrier, protecting it from the low-grade chronic inflammation involved in the development and progression of T2DM [78]. The hydrophilic/hydrophobic balance of the BA pool is also a key regulator factor for the intestinal integrity barrier. Thus, the formation of increased amounts of DCA and LCA formed from CA and CDCA, respectively, via 7α-dehydroxylation [79,80] in LD-Si rats, could have an anti-inflammatory effect, mediated, at least in part, by the inhibition of epithelial apoptosis and the promotion of barrier function [81,82]. In this regard, in vitro studies showed that LCA enhanced TNF-α-induced distribution of ZO-1, E-cadherin, occludin and claudin-1 [83]. Although we did not study the gut microbiota in this work, it is most likely that silicon ameliorated dysbiosis, among other environmental factors, such as diet, pH or the presence of enzymatic cofactors, which could influence the interactions between BA and bacteria in the gut [81]. Furthermore, silicon did not reverse the decrease in SCFA concentration found in LD rats, leading to an even lower acetate content. In vitro and in vivo studies have shown the protective effect of acetate on the intestinal barrier in metabolic syndrome animal models fed an HSF diet [84]. A reduced concentration of acetate in the cecum could be due to an increase in its absorption, leading to higher serum acetate level, which has beneficial effects in peripheral tissues. Acetate and propionate acids have been observed to stimulate the pancreatic β cell proliferation and induce insulin and primary incretin hormones (GIP and GLP1) [85]. Although butyrate levels did not change between LD and LD-Si rats, an inverse relationship of levels of apoptosis index, 12α-OHBA/non-12α-OHBA ratio, fecal cholesterol content and DTscore with butyrate content has been found (*p* < 0.05). In contrast, butyrate levels were positively correlated with silicon mucosa content (*p* = 0.001). Butyrate deprivation has been associated with low proliferation rates but increased expression of proapoptotic proteins in intestinal epithelial cells [86]. Further investigations are required to confirm the beneficial effect of silicon on the interaction between SCFA and mucosal integrity.

There are some limitations in this study that could be addressed in future research prior to clinical trials in humans. Firstly, regarding the lack of an ED group fed with Si-RM, its inclusion in the study would allow us to know the effect of Si-RM intake in an early stage of T2DM and its potential role in preventing the progression of the disease. Secondly, the microbiota in the cecum was not analyzed. Its characterization could provide further insight into the overall effect of silicon by examining the interaction between dysbiosis, metabolome and integrity of the intestinal barrier, as well as its implication in the improvement of diabetic dyslipidemia. Thirdly, there was a lack of quantifying distinct oxysterols in the cecum, whether originating from the diet or the endogenous alternative pathway. These oxysteroles act as intermediaries in the synthesis of BA, regulating FXR and LXRα/β expression in glucolipid metabolism, and may also be potentially toxic in the intestinal lumen, contributing to intestinal dysfunction. Further investigations into the role of Si-RM consumption in oxysterol signaling pathways in the intestine linked to cholesterol metabolism could be analyzed. Fourthly, it would be interesting to determine whether the antidiabetic effect of silicon is mediated by other specific pathways from FXR, such as the activation of the FXR/Takeda G protein-coupled receptor 5 (TGR5)/glucagon-like peptide-1 (GLP1) pathway, since BAs activate TGR5 in intestinal L cells, leading to the secretion of GLP1, which in turn stimulates insulin secretion from β cells. Fifthly, further studies should be carried out to verify silicon’s potential role in the liver, particularly in reversing NAFLD/NASH, which would help confirm the therapeutic targets of its cholesterol-lowering effect in the treatment of diabetic dyslipidemia.

In summary, the results presented indicate that by improving cholesterol and BA homeostasis through modulating their synthesis, absorption and excretion, Si-RM consumption significantly contributed to the reduction in plasma cholesterol levels and slowed T2DM progression. Furthermore, its ability to protect the integrity of the intestinal barrier suggests that silicon may be an effective bioactive ingredient in nutritional strategies for the treatment of diabetic dyslipidemia, either alone or in combination with antidiabetic and lipid-lowering drugs. Taken together, these findings support Si-RM consumption as a promising nutritional strategy to improve metabolic and intestinal health in patients with advanced T2DM and could serve as a precursor to potential next steps in human clinical research trials.

## 4. Materials and Methods

### 4.1. Diets and Animals

Details of the diet formulation and composition are given in Supplementary Table S1. The meat matrix (RM) was processed from lean minced pork and beef (50% each) with lard for one minute using a grinder–homogenizer connected to a 2 °C cooling bath (Stephan Universal Machine UM5, Stephan u. Söhne GmbH and Co., Hameln, Germany). After preparation, RM was freeze-dried in a LyoAlfa 10 freeze-dryer (Telstar, Terrassa, Spain) for 48 h and subsequently ground using a refrigerated mincer (Stephan Universal Machine UM5, Stephan u. Söhne GmbH and Co., Hameln, Germany) for two minutes. Each kilogram of diet contained 30% meat mixture and 70% purified formulated diet, which were mixed and sieved three times to obtain a completely homogeneous powder. Silicon-enriched meat (Si-RM) was prepared in the same way as the control meat, but with the addition of silicon. The form of silicon used in the diet was choline-stabilized orthosilicic acid (H_4_SiO_4_) obtained from Silicium organique G57TM (Glycan Group, Geneva, Switzerland). To achieve the desired dose, a certain amount of organic silicon containing 67 mg of silicon was added to 1 kg of the meat mixture. This resulted in a final silicon concentration of 20 mg per kg of the overall diet which is safe as demonstrated in previous studies [33,34,35,36,37].

As in previous studies, an advanced stage of T2DM rat model (LD group), characterized by marked dyslipidemia, hyperglycemia, significantly reduced insulin production and lower HOMA-β indexes, was used [33,35,36]. Twenty-four two-month-old male Wistar rats (Harlan S.L., Barcelona, Spain) were used. Animals were housed under controlled conditions (22.3 ± 1.9 °C, 12 h light/dark cycle) at the Animal Experimentation Center of Alcalá University, Madrid, Spain (registration number ES280050001165). Experiments were conducted in accordance with Directive 2010/63/EU on the protection of animals used for scientific purposes. This study was approved by the Advisory Committee for Science and Technology of Spain (project AGL2014-53207-C2-2-R) and by the Ethics Committee of the Complutense University of Madrid, Madrid (Spain). After a seven-day acclimatization period, animals were randomly divided into three groups. Eight rats were fed an RM-based (pork/veal, 50%/50%) high-saturated-fat high-cholesterol diet (containing 60% saturated fat, 1.4% cholesterol and 0.2% colic acid) for eight weeks to induce early-stage T2DM (ED). Sixteen rats were fed the same HSFHC for three weeks after which they were intraperitoneally administered streptozotocin (STZ, 65 mg/kg b.w.) and nicotinamide (NAD, 225 mg/kg b.w.) (both from Sigma Aldrich, Madrid, Spain), to induce late-stage T2DM (LD). As previously demonstrated, this late-onset T2DM model is appropriate for studying the impact of Si-RM intake on diabetes progression [33,34,36] and on the alterations of the intestinal barrier [87]. The ED group represents the early stage of T2DM with insulin resistance and was used as a reference group to assess the degree of improvement and retardation of diabetic dyslipidemia progression following silicon intake. After confirming fasting hyperglycemia four days later, animals were divided into two groups: the LD group, which continued on the RM and HSFHC diet until the end of the experiment, and the LD-Si group which received the Si-RM and HSFHC diet. The LD-Si group received a silicon dose of 20 mg/kg b.w./day. Tap water and food were ad libitum. Food intake and body weight were recorded daily and the cholesterol intake calculated (mg/day). To determine fecal excretion (g/day dry matter), feces were collected and weighed daily during the final week. To avoid inter-trial variations, one rat from each group was sacrificed per day. Rats were anesthetized with isoflurane (5% *v*/*v*) prior to euthanasia, and blood was extracted from the descending aorta with a heparinized syringe. The ileum, cecum and liver were dissected, weighed and processed. Cecal content was diluted with deionized water and homogenized with a Teflon homogenizer. Daily silicon excretion was measured from pooled feces obtained from the last week of the experiment for each animal, and results were expressed as silicon content excreted per day (averaged over the seven days). After centrifugation, supernatants of the cecal contents were kept in liquid N_2_ and stored at −80 °C for BA and SCFA analysis.

### 4.2. Measurement of Glycolipid Metabolic Parameters

Plasma was isolated by centrifugation for 10 min at 986× *g*, and glycemia was measured immediately using the GOD kit (Spinreact, Barcelona, Spain), at 492 nm in a plate reader (SPECTROstar Nano, BMG LABTECH, Offenburg, Germany). Insulin was measured only at the end using an ELISA kit (Rat insulin Elisa KIT, ELR-Insulin, RayBiotech, Inc., Atlanta, GA, USA). Color intensity was evaluated at 450 nm using a microplate reader (SPECTRO star Nano BMG LABTECH, Offenburg, Germany). HOMA-IR was calculated as follows: [fasting insulin (μIU/mL) × fasting glucose (mmol/L)/22.5] and HOMA-beta was calculated as follows: [20 × fasting plasma insulin (µIU/mL)]/[fasting glucose (mmol/L) − 3.5].

Triglycerides and total cholesterol were quantified using colorimetric kits (Triglycerides LQ and Cholesterol-LQ (Spinreact, Barcelona, Spain). Measurements were carried out in plate readers at 505 and 492 nm, respectively (SpectroStar Nano, BMG, LABTECH, Offenburg, Germany). LDLc and HDLc were obtained from the isolated lipoprotein fraction as described previously [35]. The atherogenic index (AI) was determined as the ratio of non-HDLc/HDLc. The diabetic trend score (DTscore) and dyslipemic score (DDscore) were calculated as follows: DTscore was obtained by considering the association of glucose, insulin and HOMA-beta, summing the tertile values of each parameter (DTscore range, 3 to 9); The diabetic dyslipemic score (DDscore) was calculated by considering the association of cholesterol, triglyceride and the AI, with the value calculated by summing the tertile values of each parameter (DDscore range, 3 to 9) [35].

### 4.3. Detection of the Amounts and Profile of BA in the Cecum Contents

The extraction of the BA from cecum samples followed previously described methods [88]. Briefly, 300 µL of H_2_O:ACN 1:1 and 15 µL of CDCA-d4 (internal standard (10 µg/mL of CDCA-d4 in H_2_O: MeOH 1:1)) were added to 200 µL of cecal content, vortexed at 1300 rpm for 30 s and left at room temperature for 10 min. The samples were vortexed again and centrifugated twice at 1300 rpm 4 °C for 5 min. Next, the supernatant was collected and evaporated under nitrogen atmosphere, reconstituted with 100 µL of H_2_O:MeOH 1:1, vortexed and centrifuged for five minutes at 1000 rpm and 4 °C. This final supernatant was analyzed, and BA quantified by HPLC-QQQ-MS. The standard curve was generated using a commercial BA mix (Sigma Aldrich, Madrid, España) that included CA, CDCA, HCA, α-MCA, TCA, GCA, DCA, GDCA, TDCA, LCA, oLCA, HDCA, UDCA at concentrations ranging from 5 to 0.001 µg/mL. The separation was carried out using a Kinetex XB-C18 100A (Phenomenex Spain, Madrid, Spain) column and a Phenomenex UHPLC C18 precolumn, ammonium acetate 2 mM in H_2_O and ACN:MeOH (1:1) as mobile phases and a constant flow rate of 1 mL/min at a temperature of 50 °C.

BA pools were defined as follows: total BA (TBA) = the sum of PBA + SBA; non-conjugated BA (cBA) = the sum of CDCA + CA + HCA + α-MCA + DCA + LCA + oLCA +UDCA + HDCA; conjugated BA (cBA) = the sum of TCA + TDCA + GCA+ GDCA; primary BA (PBA) = the sum of CDCA + CA + HCA + α-MCA + TCA + GCA; conjugated primary BA (cPBA) = the sum of TCA + GCA; secondary BA (SBA) = the sum of DCA + LCA + oLCA +UDCA + HDCA + GDCA + TDCA; conjugated secondary BA (cSBA) = the sum of TDCA + GDCA. The 12α-OHBA measured in this experiment was the sum of CA +DCA +TCA +TDCA + GCA + oLCA, while the non-12α-OHBA was αMCA + CDCA +HCA + HDCA+ UDCA+ LCA +TCDCA. The ratios of cBA/non-cBA and 12α-OHBA/non-12α-OHBA were also calculated.

### 4.4. Determination of Cecum Levels of Short-Chain Fatty Acids

The extraction of the SCFA was performed using a modified method by [89,90]. First, 300 µL of 0.5% of phosphoric acid was added to 400 µL of plasma followed by the addition of 100 µL of 4-methyl valeric acid as the internal standard and centrifuged for 15 min at 12,000 rpm and a temperature of 4 °C to facilitate protein precipitation. After centrifugation, 750 µL of supernatant was mixed with 200 µL butanol and vortexed for 30 s (×3). The supernatants were then placed in a vial with an insert for gas chromatography with flame ion detector (GC-FID) analysis. The separation was carried out using a 100% polyethylene glycol column, helium as carrier gas and a constant flow rate of 1.5 mL/min. The SCFAs were expressed in µmol/g and percentage relative to the total SCFA content.

### 4.5. Determination of Cecum and Fecal Silicon Contents

The silicon content in fecal and cecal mucosa samples was quantified using inductively coupled plasma optical emission spectrometry (ICP-OES) method, by using a high-resolution continuum source optical atomic absorption spectrometer (HS CS AAS technology, model ContrAA 700, Analytik Jena AG, Jena, Germany) and a xenon short-arc lamp (GLE, Berlin, Germany). The samples were dried over several days at a low temperature until a constant weight was achieved. Subsequently, the samples were digested using a mixture of acids, comprising nitric acid (HNO_3_), hydrogen peroxide (H_2_O_2_) and hydrofluoric acid (HF), in a high-pressure microwave system. Silicon was determined with a nitrous-oxide-acetylene-rich flame, using an individual calibration line at different concentrations, prepared from a commercial silicon standard (Certipur, 1000 mg/L silicon standardized solution from Sigma Aldrich Chemie GmbH, Steinheim, Germany). Silicon was measured at its main atomic line (251.6110 nm).

### 4.6. Histological Procedure

Sections of ileum and liver were fixed in 10% formaldehyde and embedded in paraffin, from 4 µm thick serial sections. Hematoxylin–eosin (H&E) and periodic acid–Schiff (PAS) stainings were performed for histological analysis. Images were obtained under a Leica DM LB2 light microscope and Leica DFC 320 camera (Leica, Madrid, Spain) and quantified with ImageJ software (Fiji image J; 1.52i, NIH, Bethesda, MD, USA) [87]. Villi height was measured from the tip to the base and width was measured at the base of the villi from H&E sections. Crypt depth was measured from the top of the crypt to the muscularis mucosae layer. At least 20 well-aligned villi and crypts per rat were tested. Villi height/width and villi height/crypt depth ratios were calculated. The absorptive area of the villi was estimated using the following formula: absorptive surface area = 2π × (villi width/2) × villi height. Goblet cell number per villus or crypt from PAS sections was counted with ImageJ software (1.52i, NIH, USA).

### 4.7. Immunohistochemical Assay

Ileum and liver sections from six rats per group were fixed in 4% paraformaldehyde in 0.1 M phosphate buffer, pH 7.4, and dehydrated and embedded in paraffin. After deparaffination and inactivation of endogenous peroxidase with 3% hydrogen peroxide, sections were incubated overnight at 4 °C with primary antibodies (Santa Cruz Biotechnology Quimigen, Madrid, Spain). The sections were then incubated with biotinylated secondary antibody, followed by staining with horseradish peroxidase conjugated with streptavidin–biotin, 3,3′-diaminobenzidine (DAB) and Harris hematoxylin (Sigma-Aldrich, Madrid, Spain). ImageJ 1.5.4 software (U.S. National Institutes of Health, Bethesda, MD, USA) was used to quantify immunostaining intensity for each antibody. Photographs from 10 fields per section per rat (200× magnification for image analysis) were analyzed. All slides were examined by two different researchers in a blinded manner. Protein level was expressed as immunoreactivity score (IRS) and evaluated based on staining patterns: weak (1), moderate (2), diffuse (3) or intense (4). The PCNA labeling index (PCNA-LI) was calculated as the percentage of PCNA-positive nuclei with respect to the total nuclei in the epithelium per hemicrypt.

### 4.8. Terminal Deoxynucleotidyl Transferase dUTP Nick End Labeling (TUNEL) Assay

In situ labeling of apoptotic cells was performed by identifying DNA fragmentation in paraffin-embedded sections using the terminal deoxynucleotidyl transferase dUTP nick end labeling (TUNEL) assay. After deparaffinization and rehydration, tissue sections were permeabilized with proteinase K (20 μg/mL) for 15 min at 37 °C and treated with 3% hydrogen peroxide for 5 min to quench endogenous peroxidase activity. The sections were then incubated with equilibration buffer for 10 min, followed by immediate application of TdT-enzyme working for 1 h at 37 °C. Slices were incubated with peroxidase-conjugated streptavidin and underwent subsequent staining with DAB and counterstaining with hematoxylin. The apoptotic index represents the percentage of cells undergoing apoptosis and was calculated as the ratio of TUNEL-positive cells to the total number of endothelial cells per villus, counted within 10 consecutive villi at ×10 magnification using ImageJ 1.5.4 software (U.S. National Institutes of Health, Bethesda, USA).

### 4.9. Western Blot Analysis

Total protein was extracted from the ileum and liver and tissues using lysis buffer and measured by DS protein assays (Biorad, Madrid, Spain). The extracted protein (approx. 60 μg) was separated by SDS-PAGE and transferred to PVDF membranes (Millipore, Billerica, MA, Spain). The membranes were blocked with 5% defatted milk solution at room temperature for 1 h, incubated with primary antibodies at 4 °C overnight and then incubated with the horseradish-peroxidase-conjugated secondary antibodies. Ponceau S staining (Sigma-Aldrich, Madrid, Spain) was used in the loading controls. After washing, membranes were exposed with ECL Select-kit (GE Healthcare, Madrid, Spain) and visualized using ImageQuant LAS 500 (GE Healthcare, Madrid, Spain). The quantification of the protein levels was carried out using ImageQuant 5.0 software (GE Healthcare Life Sciences).

### 4.10. Statistical Analysis

The results were expressed as mean values ± SD. Prior to statistical analysis, data were tested for homogeneity of variances using Levene’s test. For multiple comparisons, one-way ANOVA was followed by the Bonferroni test when variances were homogeneous or by the Tamhane test when variances were not homogeneous. For comparisons of non-parametric variables, the Kruskal–Wallis test was used followed by the Dunn–Bonferroni approach. Pearson’s or Spearman’s correlations between scores and parameters were also determined. Statistical significance was set at *p* < 0.05. Statistical analysis was performed using SPSS version 28.0 (SPSS Inc., Chicago, IL, USA) and graphs were drawn using GraphPad Prism version 8 (GraphPad software, Inc., La Jolla, CA, USA).

## 5. Conclusions

In conclusion, Si-RM consumption with an HSFHC diet improved the regulation of BA synthesis, enterohepatic circulation, reabsorption and excretion of cholesterol and BA, which could be involved in the previously demonstrated hypocholesterolemic effect of silicon. These beneficial changes may result from coordinate regulation of hepatic and intestinal BA metabolism through the FXR/FGF15/CYP7A1 and LXRα/β/ABCG5/8 signaling pathways, ultimately contributing to improvements in glycolipid metabolism and slowing the progression of T2DM. Furthermore, Si-RM consumption reduced the intestinal mucosa damage and repaired ileal barrier integrity by decreasing cecal TCA content and increasing hydrophilic/hydrophobic BA balance, leading to lower BA pool toxicity. Thus, consumption of Si-RM, as a functional food, may offer a novel nutritional strategy to target BA metabolism in the treatment of diabetic dyslipidemia.

## Figures and Tables

**Table 1:** Daily total and cholesterol intake, body weight increase, small intestine, liver and cecum weights, total fecal excretion, fecal fat and cholesterol contents and fecal and cecal mucosae silicon amounts of early-stage diabetes (ED), late-stage diabetes (LD) and Si-RM intake in late-stage diabetes (LD-Si) groups.

	ED Group	LD Group	LD-Si Group	*p*
Daily total intake (g/day)	17.8 ± 1.20	16.5 ± 0.55	17.1 ± 0.55	NS
Daily cholesterol intake (mg/day)	3.6 ± 0.36 ^a^	154.0 ± 12.1 ^b^	167.1 ± 8.5 ^b^	<0.0001
Body weight increase (g)	134.4 ± 25	117.3 ± 32.3	127.6 ± 34.6	NS
Small intestine weight (g)	1.86 ± 0.16	1.90 ± 0.27	1.86 ± 0.21	NS
Liver weight (g)	10.34 ± 0.82 ^a^	18.43 ± 2.38 ^b^	17.43 ± 2.42 ^b^	<0.001
Cecum weight (g)	2.39 ± 0.57	2.49 ± 0.48	2.64 ± 0.73	NS
Cecal content (g)	1.59 ± 0.61	1.63 ± 0.38	1.59 ± 0.41	NS
Cecal mucosae silicon (µg/g)	1.14 ± 0.25 ^a^	0.76 ± 0.12 ^b^	0.67 ± 0.18 ^b^	<0.0001
Daily fecal excretion (g dry matter)	1.14 ± 0.08 ^a^	1.60 ± 0.06 ^b^	1.96 ± 0.24 ^c^	<0.0001
Fecal fat (mg/g dry matter)	80.0 ± 6.7 ^a^	170.6 ± 10.1 ^b^	254.0 ± 14.3 ^c^	<0.0001
Fecal Cholesterol (mg/g dry matter)	1.43 ± 0.49 ^a^	13.23 ± 0.62 ^b^	22.30 ± 4.64 ^c^	<0.0001
Fecal silicon (mg/g dry matter)	2,82 ± 0.19 ^a^	1.54 ± 0.42 ^b^	3.26 ± 0.88 ^a^	<0.0001
Daily silicon excretion (µg/g dry matter)	2.95 ± 0.86 ^a^	2.73 ± 0.84 ^a^	6.42 ± 2.04 ^b^	<0.001

Values expressed as mean ± SD. Different letters (a < b < c) indicate significant differences between groups (*p* < 0.05, ANOVA followed by Bonferroni post hoc test). NS: No significant differences between groups.

**Table 2:** Plasma glucose, insulin, triglyceride and cholesterol concentrations, HOMA-IR, HOMA-beta, atherogenic index (AI), DTscore and DDscore of early-stage diabetes (ED), late-stage diabetes (LD) and Si-RM intake in late-stage diabetes (LD-Si) groups.

	ED Group	LD Group	LD-Si Group	*p*
Glucose (mmol/L)	13.92 ± 0.91 ^a^	18.11 ± 1.65 ^b^	15.26 ± 2.07 ^a^	<0.001
Insulin (μUI/mL)	15.84 ± 0.73 ^a^	5.41 ± 1.23 ^b^	8.11 ± 1.79 ^c^	<0.0001
HOMA-IR ^(a)^	9.79 ± 0.64 ^a^	4.57 ± 1.55 ^b^	5.87 ± 1.5 ^b^	<0.001
HOMA-beta ^(b)^	31.13 ± 3.33 ^c^	7.24 ± 1.35 ^a^	12.75 ± 3.04 ^b^	<0.001
Triglycerides (mmol/L)	1.79 ± 0.25 ^c^	0.88 ± 0.11 ^b^	0.60 ± 0.16 ^a^	<0.001
Cholesterol (mmol/L)	2.05 ± 2.76 ^a^	2.79 ± 2.04 ^b^	2.24 ± 1.35 ^c^	<0.0001
AI ^(c)^	0.43 ± 0.24 ^a^	1.68 ± 0.55 ^c^	1.04 ± 0.16 ^b^	<0.001
DTscore ^(d)^ (3 to 9)	3.29 ± 0.49 ^a^	8.57 ± 0.79 ^c^	6.14 ± 0.90 ^b^	<0.001
DDscore ^(e)^ (3 to 9)	5.29 ± 0.95 ^a^	7.71 ± 1.25 ^b^	5.14 ± 0.90 ^a^	<0.001

Values expressed as mean ± SD. Different letters (a < b < c) indicate significant differences between groups (ANOVA, Bonferroni post hoc test, *p* < 0.05). NS: Non-significant differences between groups. ^(a)^ HOMA-IR, homeostatic model assessment of insulin resistance: [fasting glucose (mmol/L) × fasting insulin (µIU/mL)]/22.5; ^(b)^ HOMA-beta: 20 × fasting insulin (µIU/mL)/[fasting glucose (mmol/L) − 3.5]; ^(c)^ AI, Atherogenic index: total cholesterol/HDLc; ^(d)^ DTscore, Diabetes trend score: Glucose score + Insulin score + HOMA-beta score; ^(e)^ DDscore, Dyslipidemic Diabetes score: Triglyceride score + Cholesterol score + Atherogenic index score.

## Data Availability

Data are available upon request to the authors. The data are not publicly available due to principle of confidentiality.

## Supplementary Materials

The following supporting information can be downloaded at: https://www.mdpi.com/article/10.3390/ijms252111405/s1.
