# Supplementary material for: Silicon-Enriched Meat Ameliorates Diabetic Dyslipidemia by Improving Cholesterol, Bile Acid Metabolism and Ileal Barrier Integrity in Rats with Late-Stage Type 2 Diabetes
Source: Int J Mol Sci. 2024 Oct 23;25(21):11405. doi: 10.3390/ijms252111405 (PMC11547133; doi:10.3390/ijms252111405)
Supplement: Supplementary file 1 [file ijms-25-11405-s001.zip › ijms-3241327-supplementary.pdf]

Table S1. Experimental diets of early-stage diabetes (ED), late-stage diabetes (LD), and late-stage diabetes-silicon (LD-Si) groups.

Dietary components	ED diet	LD diet	LD-Si diet
**Nutrient composition**			
Proteins (% En[(a)])	14. 2	14.0	14.0
Meat Fat (% En)	49.4	49.0	49.0
SFA[(b)]/MUFA[(c)]/PUFA[(d)] ratio	2.7/3.0/1.0	2.1/2.3/1.0	2.1/2.3/1.0
Cholesterol (%)	0.02	0.93	0.93
Energy content (MJ/kg)	20.13	20.31	20.31
**Ingredients (g/kg)**			
Sucrose	68.25	68.25	68.25
Corn starch	286.73	275.73	275.71
Casein	94.25	94.25	94.25
Maltodextrin	94.25	94.25	94.25
Cellulose	48.86	48.86	48.86
PM 205B SAFE[(e)]	50.05	50.05	50.05
PV 200 SAFE[(f)]	7.15	7.15	7.15
Soybean oil	47.91	47.91	47.91
L-Cysteine	2.02	2.02	2.02
Cholesterol	0	9.1	9.1
Cholic acid	0	1.3	1.3
Silicon	0	0	0.02
Lyophilized restructured meat	301.14	301.14	301.14

ED: Early-stage diabetic control group, diet containing control restructured meat; LD: Late-stage diabetic group, diet containing control restructured meat, 1.4% cholesterol and 0.2% cholic acid; LD-Si: Late-stage diabetic silicon-enriched meat group. (a) En: Total energy; (b) SFA: saturated fatty acids; (c) MUFA: monounsaturated fatty acids; (d) PUFA: polyunsaturated fatty acids; (e) PM 205B SAFE: mineral mix; (f) PV 200 SAFE: vitamin mix. *Calculated data considered energy equivalents for carbohydrates 16.73 kJ/g (4.0 kcal/g), fat 37.65 kJ/g (9.0 kcal/g), and protein 16.73 kJ/g (4.0 kcal/g).
